# Navigating the molecular landscape: integrated multiomics liquid biopsy for biomarker discovery in early detection and monitoring of colorectal cancer

**DOI:** 10.3389/fmolb.2026.1795133

**Published:** 2026-03-18

**Authors:** Xuanqiang Fan, Jinyu Shi, Yiwan Shang, Hui Xu

**Affiliations:** 1 The Third Affiliated Hospital of Henan University of Traditional Chinese Medicine, Zhengzhou, China; 2 School of Acupuncture and Massage, Henan University of Traditional Chinese Medicine, Zhengzhou, China; 3 School of Chinese Medicine, Henan University of Traditional Chinese Medicine, Zhengzhou, China

**Keywords:** artificial intelligence, bioinformatics, colorectal cancer, liquid biopsy, molecular markers, multiomics

## Abstract

The elevated mortality associated with colorectal cancer is largely due to delayed diagnosis and post-treatment disease recurrence, highlighting the urgent clinical need for molecular markers with exceptional sensitivity and specificity to support early detection and longitudinal disease monitoring. Although conventional liquid biopsy methods targeting single analytes have clinical value, they have inherent limitations in terms of early screening sensitivity, specificity, and tissue-of-origin identification. This review systematically catalogs multiomics biomarker discoveries and summarizes integration strategies for liquid biopsy in colorectal cancer, highlighting how the combination of genomic, epigenomic, transcriptomic, proteomic, and metabolomic signals can improve early detection, MRD monitoring, and treatment guidance. By synthesizing the existing literature, we focus on how this integrated approach overcomes the constraints of single-signal detection, comprehensively delineates the molecular landscape of colorectal cancer, and advances the development of high-performance multiomics biomarker panels. Furthermore, this review explores recent progress in the application of bioinformatics and artificial intelligence-driven cross-omics integration models to optimize biomarker panel performance. In summary, this comprehensive analysis of multiomics integration not only clarifies approaches to molecular marker discovery but also provides a theoretical basis for refining clinical management strategies for colorectal cancer, thereby establishing a framework for precision oncology practices built on continuous molecular surveillance.

## 1 Introduction

Colorectal cancer (CRC) is one of the most common and deadly malignancies worldwide, and its unfavorable prognosis is largely attributable to late-stage diagnosis and post-treatment recurrence and metastasis (Siegel et al., 2024). Although colonoscopy is considered the diagnostic gold standard, its invasive nature, high cost, and the need for extensive bowel preparation substantially limit its acceptability and scalability in population-based screening programs (Shahini et al., 2023). In addition, traditional serum tumor markers such as carcinoembryonic antigen (CEA) have suboptimal sensitivity and specificity, making them inadequate for early detection and longitudinal disease monitoring.

**FIGURE 1:**
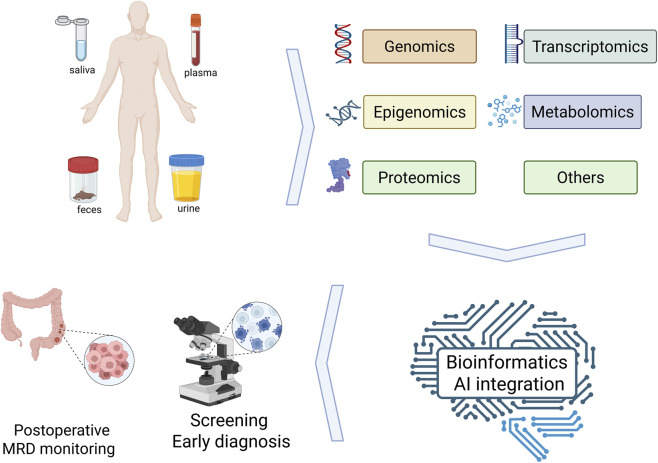
Schematic overview of multiomics liquid biopsy in colorectal cancer. Schematic overview of multiomics liquid biopsy in colorectal cancer, illustrating different sample types (saliva, plasma, feces, urine), major omics layers (genomics, transcriptomics, epigenomics, proteomics, metabolomics and others), and the bioinformatics/AI integration pipeline (quality control, normalization, feature selection and multi-omics modeling), which ultimately supports clinical applications such as screening and early diagnosis as well as postoperative minimal residual disease (MRD) monitoring.

In recent years, the emergence of liquid biopsy technologies has created new opportunities for precision management of CRC by capturing and analyzing tumor-derived biomolecules circulating in body fluids, thereby providing valuable real-time information for early diagnosis and treatment response assessment (Ma et al., 2024). However, early liquid biopsy strategies that rely on single-type biomarkers have gradually revealed intrinsic limitations in detection sensitivity, tissue-of-origin identification, and elucidation of resistance mechanisms. With the rapid advancement of high-throughput technologies, the integration of multiple omics layers—each requiring distinct analytical tools and platforms—has expanded beyond purely genomic analyses to encompass epigenomic, transcriptomic, proteomic, and metabolomic dimensions. Such multiomics integration strategies transcend the scope of traditional approaches, transforming liquid biopsy from a simple diagnostic tool into a powerful platform for biomarker discovery (Dar et al., 2023). By integrating complementary information across multiple molecular layers, these approaches not only enable cross-validation to enhance result robustness but also provide opportunities to uncover core signaling pathways and feedback mechanisms that drive tumor progression. Against this backdrop, this review aims to systematically delineate how integrated multiomics liquid biopsy can be leveraged to discover and validate next-generation biomarkers, and to outline future directions, with the ultimate goal of promoting further paradigm shifts in precision diagnosis and treatment of CRC (Figure 1).

## 2 Current status of single-omics study on CRC liquid biopsy

### 2.1 Genomics

Circulating tumor DNA (ctDNA), the central analyte in liquid biopsy, originates predominantly from tumor cell apoptosis, necrosis, or active secretion and carries tumor-specific somatic mutations, providing a key molecular basis for the early detection and dynamic monitoring of CRC (De Paolis et al., 2025). Current mainstream ctDNA detection technologies can be broadly classified into two categories, the first of which comprises amplification-based targeted methods (García-Foncillas et al., 2017). Techniques such as droplet digital polymerase chain reaction (ddPCR) enable highly sensitive, absolute quantification of known hotspot mutations (e.g., KRAS G12D/V, inactivating APC mutations), with limits of detection down to 0.01% variant allele frequency (VAF), and are well suited for single-gene dynamic monitoring (Xu D. et al., 2023). However, ddPCR is inherently restricted to predefined variants and is therefore not suitable for comprehensive screening of APC, which harbors numerous truncating mutations distributed across the entire coding region. The second category comprises high-throughput sequencing technologies, including amplicon-based targeted sequencing, hybrid capture-based targeted sequencing (e.g., Cancer Personalized Profiling by Deep Sequencing, CAPP-Seq) and whole-genome sequencing (WGS), which can concurrently detect multiple driver mutations, copy number variations, and tumor mutation burden, thereby providing a more comprehensive picture of tumor heterogeneity (Li et al., 2019; Nikanjam et al., 2022).

In the genomic application of liquid biopsy for CRC, early detection efforts primarily focus on tracking frequent somatic driver mutations, particularly in tumor suppressor genes such as APC and TP53, as well as FBXW7, KRAS, ERBB2, BRAF, PIK3CA, and SMAD4 (Hall and Benndorf, 2022; Jafari et al., 2022; Mannucci and Goel, 2024; Tock et al., 2025). However, under conditions of low tumor burden at early stages, the abundance of tumor-specific mutations in ctDNA usually constitutes less than 1% of total circulating cell-free DNA and is easily obscured by a large background of normal DNA. At such low variant allele frequencies, true tumor variants compete with technical noise arising from PCR and sequencing errors, as well as biological background alterations from clonal hematopoiesis and age-related somatic mutations in normal tissues, making reliable discrimination challenging even with ultra-deep NGS. In addition, tumor- derived cfDNA fragments show characteristic size and end-motif patterns that can be diluted within the predominant pool of non-tumor cfDNA, and the short half-life (∼1–2 h) of ctDNA further narrows the temporal window for capturing these rare events, collectively limiting early detection rates (Dasari et al., 2020). Moreover, existing technologies have inherent limitations: targeted amplification methods can only detect known mutations and fail to capture novel variants, whereas high-throughput sequencing is constrained by high cost and analytical complexity, hindering its widespread adoption in large-scale population screening.

### 2.2 Epigenomics

Epigenomic studies have primarily focused on alterations in DNA methylation and histone modifications. In particular, aberrant CpG promoter methylation, characterized by strong tissue specificity, stable occurrence at the precancerous adenoma stage, and relative enrichment in ctDNA, represents an attractive molecular target for liquid biopsy (Li et al., 2024). DNA methylation analysis is the central strategy in current epigenomic research, with major methods including bisulfite conversion coupled with methylation-specific PCR, Methyl-CpG binding domain sequencing, whole-genome bisulfite sequencing (WGBS) (Tan et al., 2024), and high-throughput methylation arrays based on Illumina platforms (Mannens et al., 2022). While WGBS (and SMRT) provides unbiased, genome-wide coverage of CpG islands and non-island regions for methylation biomarker discovery, targeted methylation sequencing focuses high-depth, cost-effective profiling on a limited set of clinically relevant loci. Although this complementary strategy links global landscape mapping with practical implementation, its application is still largely confined to exploratory research (Zhu et al., 2025), whereas current clinical methylation testing predominantly relies on arrays or targeted panels.

With respect to biomarkers, the methylation of *SEPT9* and *SDC2* represents a relatively mature molecular marker for CRC screening (Chilimoniuk et al., 2025), and a study including 83 patients with CRC reported that a methylation panel comprising SEPT9, SDC2, and VIM achieved a sensitivity of 91.4% and a specificity of 100% (AUC = 0.99) for CRC diagnosis after model validation (Liu et al., 2024). Additional genes, such as *PRDM12*, *FOX1*, *KCNQ5*, and *C9orf50*, have also been identified to have diagnostic specificity (Cao et al., 2020; Yun et al., 2025).

Despite the high enrichment of SEPT9 methylation in CRC, positive signals can also occur in other malignancies, such as breast and cervical cancers, thereby limiting its utility in multi-cancer differential diagnosis (Jędrzejczak et al., 2024). Furthermore, a meta-analysis demonstrated that SEPT9-based methylation assays performed less favorably than traditional fecal immunochemical tests in asymptomatic individuals (Song et al., 2017).

### 2.3 Transcriptomics

Liquid biopsy strategies based on transcriptomics primarily focus on circulating RNA species, including mRNA, microRNA (miRNA), long non-coding RNA (lncRNA), circular RNA (circRNA), and exosomal RNA (Raza et al., 2022; Behrouzian Fard et al., 2025). Mainstream detection technologies encompass PCR-based methods, high-throughput sequencing, and microarrays; PCR-based approaches include ddPCR and RT-qPCR, while sequencing-based approaches include RNA sequencing and targeted RNA sequencing.

Transcriptomic biomarkers for early CRC liquid biopsy have formed a multidimensional marker system centered on non-coding RNAs, with coding RNAs and RNA modifications as important complements, among which miRNAs are the most extensively studied; plasma levels of miR-143-3p, miR-21, miR-29a, miR-92a, and miR-155 are elevated in patients with CRC (Luo et al., 2013; Pająk et al., 2025; Wang X. et al., 2025). Several miRNAs, such as miR-29a, miR-92a, miR-223, miR-29a-3p, and miR-186-5p, can also be detected in fecal or saliva samples and show potential for assisting in CRC and precancerous lesion screening (Behrouzian Fard et al., 2025). In a study including 151 patients with CRC and 160 healthy controls, the lncRNA HIF1A-AS1 was found to be significantly elevated in the serum of patients with CRC (AUC = 0.960) (Gong et al., 2017), and exosome-derived FOXD2-AS1, NRIR, and XLOC_009459 were elevated in the serum of patients with early-stage CRC (AUC = 0.743/0.660/0.689) (Yu et al., 2021). CircRNAs possess greater resistance to degradation and higher stability, making them highly attractive biomarkers for cancer detection. The specific elevation of circ_0004771 in the plasma of patients with CRC and its decline after surgery suggest that circ_0004771 has both diagnostic and prognostic monitoring value (Pan et al., 2019). These single biomarkers are often combined into panels to further optimize diagnostic performance; for instance, a panel comprising miR-144-3p, miR-425-5p, and miR-1260b achieved a sensitivity of 93.8% and a specificity of 91.3% (Tan et al., 2019).

Despite these advances, transcriptomics-based liquid biopsy faces multiple challenges. The absolute abundance of circulating free RNA (cfRNA) is extremely low, and cfRNA is easily contaminated by microbial, environmental, and cellular RNAs.Residual cells (e.g., platelets and other blood cells) during plasma preparation can release cellular RNA and DNA, which significantly confound the detection and quantification of cell-free RNA (cfRNA); in addition, conventional random-primed reverse transcription approaches introduce inherent biases, including a preference for longer transcripts, the loss of short RNAs (sequence/length-related bias) and high susceptibility to DNA contamination, which further impair the coverage representation and accurate quantification of cfRNA transcripts. Although innovative techniques such as polyA-tailing-based sequencing have partially alleviated these issues, pre-analytical variables remain difficult to standardize. The combination of high-dimensional data and limi ted sample sizes predisposes models to overfitting, and clinical validation to date has largely relied on small-scale studies (Barbitoff et al., 2020; Loy et al., 2024).

### 2.4 Proteomics

In proteomics-based liquid biopsy for early CRC detection, mainstream approaches include mass spectrometry-based untargeted strategies and targeted proteomics. Common mass spectrometry methods such as data-independent acquisition and parallel reaction monitoring enable hypothesis-free proteomic profiling and are therefore preferred tools for early-stage biomarker discovery. Targeted strategies encompass antibody and antigen arrays, proximity extension assays, and reverse-phase protein arrays, among others (Ding et al., 2022). In addition, highly sensitive immunoassay platforms, such as Olink and Luminex multiplex systems, are widely used for validating candidate proteins in large cohorts (Cui et al., 2022). Exosomes, as important carriers of tumor-derived circulating proteins, are typically enriched by size-exclusion chromatography or immunoaffinity capture (Sidhom et al., 2020; Kaddour et al., 2021), followed by functional characterization using ELISA or nano-flow cytometry. Zhang et al. (Zhang et al., 2024) developed a method termed DSPE-beads for extracellular vesicle (EV) isolation, which offers high detection efficiency and low cost, providing robust technical support for systematic screening of early CRC protein biomarkers.

With respect to core protein biomarkers, traditional indicators, such as CEA and carbohydrate antigen 19-9 (CA19-9) are widely used for postoperative surveillance but have limited sensitivity and specificity, rendering them inadequate for early screening (Hu et al., 2022). Recent studies using machine learning and clinical validation have identified several novel proteins with high diagnostic performance. For example, the “ColonTrack” model incorporating CTTN, HNRNPK, and PSMC6 achieved an AUC of 0.974 (sensitivity = 0.923, specificity = 0.947) in an external validation cohort of 274 CRC and non-CRC subjects (Wang J. et al., 2025). Hua et al. (Hua et al., 2024) developed a seven-protein panel (LRG1, C9, IGFBP2, CNDP1, ITIH3, SERPINA1, ORM1) that, in blinded validation, achieved a sensitivity of 81.5%, specificity of 97.9%, and overall accuracy of 92.0%, with a sensitivity of 49% for detecting advanced precancerous lesions. For lesions larger than 1.5 cm, the detection rate approached 60%, and for high-risk polyps, such as tubulovillous adenomas, the sensitivity reached 100%, highlighting the panel’s considerable utility for early CRC screening and precancerous lesion warning. These biomarkers reflect the secretory characteristics of tumor tissues and are valuable for both early diagnosis and dynamic disease monitoring.

The main limitations of proteomics center on the complex background of bodily fluids and the technical challenges in exosome isolation. High-abundance plasma proteins (e.g., albumin) can severely mask low-abundance tumor-related proteins, and the protein cargo of EVs in plasma only partially overlaps with that in tumor tissue, with tissue-derived EVs containing more tumor-specific information (Wang J. et al., 2025). Furthermore, the vast majority of proteomics-based CRC liquid biopsy diagnostic models remain in the research stage. The transition from laboratory-developed tests to commercially available *in vitro* diagnostic products approved by regulatory authorities is lengthy and fraught with challenges.

### 2.5 Metabolomics

Metabolomics-based liquid biopsy for early CRC detection primarily relies on liquid chromatography-mass spectrometry (LC-MS), gas chromatography-mass spectrometry, and nuclear magnetic resonance (NMR) technologies, and can be classified into untargeted and targeted metabolomics according to analytical strategy, both of which are applicable to plasma, serum, and urine samples (Zhang et al., 2014; Deng et al., 2019; Kim et al., 2019). Untargeted strategies can comprehensively profile all detectable metabolites and are suitable for identifying differential metabolites in early CRC. Targeted strategies perform precise quantitative analyses of metabolites with known structures and are used to validate early-screened differential metabolites and construct diagnostic models, often forming the basis of commercial testing platforms.

Deng et al. (2019) employed a targeted LC-MS approach to profile 140 metabolites and identified a model (Model III) comprising only diacetylspermine and kynurenine, which exhibited the smallest decline in AUC from training to testing sets, thereby confirming the robustness of the selected biomarkers. At a specificity of 80%, Model III achieved a sensitivity of 80.0% in the training set and 74.0% in the testing set. Kim et al. (2019) used urine NMR-based metabolomics and identified taurine (AUC = 0.823), alanine (AUC = 0.783), and 3-aminoisobutyrate (AUC = 0.842) as candidate biomarkers for early CRC detection. Kuwabara et al. (2022) found that lactate and pyruvate levels were elevated in saliva samples from patients with CRC compared with healthy controls.

Metabolomics reflects functional states through the measurement of small-molecule metabolites, but its limitations stem from the inherent features of metabolic processes. Metabolite levels are strongly influenced by non-tumor factors such as diet, physical activity, gut microbiota, and hepatic and renal function, resulting in large inter-individual variability and dynamic changes with physiological state, which complicate the establishment of stable diagnostic thresholds. More importantly, many metabolic alterations represent downstream consequences rather than drivers of tumorigenesis, which may limit their value for the early warning of precancerous lesions. Different analytical platforms (e.g., MS vs. NMR) have distinct detection biases, further increasing the difficulty of achieving comprehensive metabolite coverage and cross-platform standardization (Schmidt et al., 2021).

## 3 Integration logic and technical framework of multiomics liquid biopsy

CRC initiation and progression involve coordinated dysregulation across multiple layers—including genomic alterations, epigenetic regulation, transcriptional reprogramming, protein expression, and metabolic remodeling, forming a complex network (Stefani et al., 2021; Li et al., 2024). Single-omics technologies can capture only a fragment of this network and cannot systematically reveal the core pathways and dynamic interactions that drive tumor progression. Although some single-omics panels have reported AUC values approaching or exceeding 0.95 in small cohorts, their reproducibility and generalizability in real-world settings remain limited. More importantly, in ultra-low tumor burden settings, such as stage I CRC, population screening, detection of precancerous lesions, and postoperative minimal residual disease (MRD) monitoring, signals from a single molecular layer still suffer from limited sensitivity and are easily confounded by non-tumor factors, representing inherent structural bottlenecks. These structural bottlenecks, including extremely low tumor-derived signal fractions within abundant normal background, short biomarker half-lives and assay detection limits, affect genomic, epigenomic, transcriptomic, proteomic and metabolomic biomarkers in the same clinical environment. Therefore, integrating multiomics data is an inevitable direction for overcoming current bottlenecks and achieving more accurate early detection, response assessment, and prognostic evaluation. Multiomics liquid biopsy overcomes the “dimensional deficiency” of single-omics approaches by leveraging diverse integration strategies and bioinformatics (Matsuoka and Yashiro, 2024), as well as artificial intelligence (AI) technologies (He et al., 2023). By integrating molecular signals from the genome, epigenome, transcriptome, proteome, and metabolome, a more comprehensive molecular regulatory network for CRC can be constructed (Zhou K. et al., 2025). The ultimate goal of multiomics integration is not simply to stack data, but to reveal intrinsic connections among molecular events based on CRC-related mechanisms, through coordinated signal analysis (Gao et al., 2024). This enables a transition from “fragmented molecular testing” to “system-level molecular interpretation,” providing a more comprehensive molecular basis for the early diagnosis, dynamic monitoring, and precise treatment of CRC.

The Table 1 summarizes single-omics liquid biopsy approaches in CRC, comparing genomics, epigenomics, transcriptomics, proteomics, and metabolomics with respect to detection targets, representative techniques, example biomarkers, and key advantages and limitations.

**TABLE 1:** Comparison of single-omics liquid biopsy approaches in colorectal cancer.

Omics	Primary analytes	Representative technologies	Example biomarkers/panels	Main limitations	Refs
Genomics	ctDNA mutations	ddPCR; targeted NGS (e.g., CAPP-Seq); WGS	e.g., APC, KRAS, TP53, BRAF	Low variant allele frequency (VAF) in early-stage disease; high sequencing cost for broad panels	García-Foncillas et al. (2017), Li et al. (2019), Hall and Benndorf (2022), Nikanjam et al. (2022), Xu et al. (2023a)
Epigenomics	DNA methylation	MSP, targeted methylation sequencing, WGBS, arrays	e.g., mSEPT9, SDC2, KCNQ5	Potential cross-cancer positivity for some markers; assay- and platform-dependent variability	Jędrzejczak et al. (2024), Tan et al. (2024), Chilimoniuk et al. (2025), Zhu et al. (2025)
Transcriptomics	miRNA, lncRNA, circRNA, exosomal RNA, etc.	RT-qPCR, ddPCR, RNA-seq	e.g., miR-21, miR-29a, HIF1A-AS1, circ_0004771	Low abundance in circulation; strong sensitivity to pre-analytical conditions	Luo et al. (2013), Pan et al. (2019), Raza et al. (2022), Loy et al. (2024), Behrouzian Fard et al. (2025)
Proteomics	Circulating/exosomal proteins	Mass spectrometry immunoassay	e.g., CEA, CA19-9, CTTN, HNRNPK, PSMC6	Masking of low-abundance tumor proteins by high-abundance plasma proteins; technical challenges in EV isolation	Sidhom et al. (2020), Cui et al. (2022), Hua et al. (2024), Wang et al. (2025a)
Metabolomics	Small-molecule metabolites	LC-MS, GC-MS, NMR	e.g., diacetylspermine, kynurenine, taurine, lactate	Strongly influenced by diet, microbiome, and systemic physiology	Zhang et al. (2014), Deng et al. (2019), Kim et al. (2019), Schmidt et al. (2021)

Comparison of singleomics liquid biopsy approaches in colorectal cancer, summarizing for each omics layer (genomics, epigenomics, transcriptomics, proteomics and metabolomics) the primary analytes, representative technologies, example biomarkers or panels, and the main limitations that may affect clinical translation.

### 3.1 Core logic of multiomics integration

The primary objective of multiomics integration is to enhance detection sensitivity through complementary information. In low-tumor-burden settings, such as early detection, the limitations of single-omics biomarkers are particularly evident. For example, ctDNA point mutation-based assays may miss early tumors because of extremely low VAF, requiring repeated sampling to improve accuracy (Wan et al., 2017). Although methylation assays targeting specific genes (such as *SEPT9*) can improve sensitivity, their direct link to clonal evolution is relatively weak, and they can be influenced by benign lesions (Khabbazpour et al., 2024; Li and Sun, 2024), whereas traditional protein markers such as CEA lack specificity. The core logic of multiomics integration lies in multidimensional cross-validation and signal amplification. By analyzing multiple molecular types in parallel from the same or different biofluids of a patient, tumor signals can be captured from multiple independent yet interrelated biological layers. For instance, CEA and CA19-9 have low sensitivity in early CRC. Lu et al. (Lu et al., 2022) analyzed plasma and serum samples from newly diagnosed patients with CRC and healthy individuals for mSEPT9, CEA, and CA19-9, and found that combined detection improved sensitivity (78.43%), specificity (86.07%), and AUC (0.878) compared with any single marker.

Higher-level multiomics integration aims to clarify causal and regulatory networks across molecular layers, thereby identifying biomarker combinations with greater biological and clinical relevance. CRC development involves multiple molecular pathways. The classical chromosomal instability (CIN) pathway usually follows an adenoma-carcinoma sequence, characterized by APC mutations (leading to sustained activation of Wnt/β-catenin signaling), subsequent KRAS mutations (activating the MAPK pathway), and late TP53 inactivation. In contrast, the CpG island methylator phenotype (CIMP) pathway, especially that associated with serrated lesions, is characterized by extensive hypermethylation of CpG islands in multiple gene promoters, often leading to epigenetic silencing of Wnt inhibitors, such as the SFRP family. These two molecular subtypes also differ mechanistically: in CIN-type tumors, Wnt and EGFR pathway abnormalities mainly arise from gene mutations, ligand overexpression, or receptor amplification, whereas CIMP-type tumors are more often driven by epigenetic dysregulation. Notably, Wnt pathway activation blocks β-catenin phosphorylation and subsequent ubiquitin-proteasome-mediated degradation, resulting in its accumulation and nuclear translocation for transcriptional activation, which is mechanistically distinct from EGFR signaling that relies on receptor tyrosine kinase phosphorylation cascades (Duta-Ion et al., 2024; Gharib and Robichaud, 2024; Yang et al., 2024). Through multiomics integration, one can construct longitudinal regulatory maps spanning “genomic alterations → epigenetic modifications → transcriptomic dysregulation → protein functional changes.” For example, integrated analyses can reveal associations between APC loss or mutation, DNA hypomethylation, activation of Wnt target genes, such as MYC, and elevated circulating c-Myc protein levels (Mármol et al., 2017). Such mechanism-driven integration not only supports the identification of superior panels that improve early CRC detection, but also deepens understanding of tumor subtype heterogeneity and resistance mechanisms, thereby elevating liquid biopsy from a mere “testing tool” to a “window into disease mechanisms.”

### 3.2 Mainstream multiomics integration models

**FIGURE 2:**
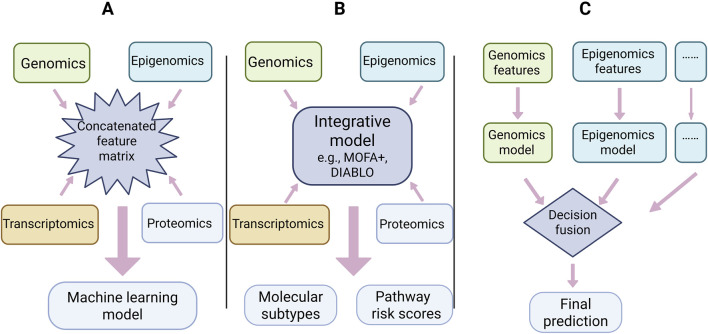
Schematic illustration of early, intermediate and late multiomics integration strategies. **(A)** Early integration: Omics-specific data matrices (genomics, epigenomics, transcriptomics, proteomics) are concatenated into a single high-dimensional feature matrix, which is then directly used to train a machine learning model. This feature-level fusion is conceptually simple but can be sensitive to scale differences, high-dimensional noise and feature redundancy. **(B)** Intermediate integration: Each omics layer is first projected into a shared latent space by an integrative model (e.g., MOFA+, DIABLO), enabling cross-omics pattern discovery before deriving molecular subtypes or pathway-level risk scores. This representation-level fusion balances information sharing and dimensionality reduction. **(C)** Late integration: Separate predictive models are trained for each omics layer, and their outputs are combined at the decision level (e.g., weighted voting or stacking) to generate the final prediction. This decision-level fusion is modular and clinically flexible, but may miss low-level cross-omics interactions captured by earlier integration schemes.

Multiomics integration is not a single strategy. Based on the timing and logical level of data fusion, Rappoport and Shamir (Rappoport and Shamir, 2018) classified integration methods into three categories: early, intermediate, and late integration. However, in practice, these categories are not mutually exclusive (Figure 2).

Early integration is the most straightforward strategy, in which raw feature matrices from different omics are concatenated into a single “sample-feature” matrix, and then conventional algorithms such as K-means or hierarchical clustering are applied. This approach operates at the feature level and does not account for distributional differences or scale mismatches among omics datasets. In CRC studies, early integration may be used for rapid exploratory analyses, such as combining ctDNA mutation sites, methylation β-values, and concentrations of a few serum protein markers. However, this strategy faces inherent data heterogeneity: data types such as ctDNA VAFs on the order of 10^−4^ to 10^−3^ and serum protein concentrations in the ng/mL range (Dao et al., 2023; Guszcz et al., 2025) differ greatly in signal type and noise structure. Under such conditions, simple concatenation tends to allow high-variance or high-background features to dominate the analysis, potentially masking low-abundance driver events.

Intermediate integration aims to build a unified model that jointly represents all omics datasets, and is currently central and cutting-edge in multiomics analysis. Technically, it can be subdivided into: (i) similarity-based integration, such as DIABLO (Singh et al., 2019) and Mintea (Muller et al., 2024) which use extended sparse generalized canonical correlation analysis to identify cross-omics co-varying feature modules and can be applied to dissect host–microbiome interactions in CRC; (ii) statistical-model-based integration, exemplified by multiomics factor analysis (MOFA+) (Argelaguet et al., 2018; Argelaguet et al., 2020) which uses a Bayesian framework to project data into shared latent spaces, thereby discovering molecular subtypes in an unsupervised manner; (iii) (iii) emerging knowledge-guided deep integration, such as DeePathNet (Cai et al., 2024), which incorporates known cancer pathway information as prior knowledge and employs Transformer architectures for end-to-end joint modeling, markedly improving predictive accuracy and interpretability. Intermediate integration can reveal deep cross-omics mechanisms, but also faces challenges such as high computational complexity and sensitivity to algorithm design and data quality. Choosing an integration strategy requires balancing the specific clinical questions, data properties, and demands for interpretability *versus* predictive performance.

Late integration refers to clustering or modeling each omics dataset separately and then combining the resulting outputs into a single consensus solution. This approach respects the intrinsic characteristics of each omics dataset and allows the use of the most appropriate algorithms and distance metrics for each dataset. Multiomics Late Integration (MOLI) (Sharifi-Noghabi et al., 2019) is a representative method that uses a deep neural network framework with modality-specific encoders to learn feature representations from somatic mutation, copy number, and gene expression data, which are then concatenated and jointly optimized in deeper layers to predict drug responses. This architecture allows the model to leverage the strengths of independent analyses for each modality while achieving decision-level fusion, and has shown superior predictive performance compared with early integration in external validations. In CRC liquid biopsy-based clinical decision-making, the logic of late integration is highly practical. For example, combining ctDNA-based micro-lesion detection with risk stratification derived from circulating tumor cell counts can provide strong consensus evidence for recurrence risk when both indicate high risk. The advantages of this approach are flexibility and robustness; it is easy to integrate the results from different technical platforms, and each module can be independently optimized and validated. Its limitation is the potential loss of deep associations present at the raw feature level across omics.

Together, early, intermediate, and late integration form a relatively complete methodological spectrum. In real-world applications of CRC early detection and monitoring, intermediate integration has become a methodological focus owing to its capacity to uncover biological mechanisms, while late integration shows great translational potential because of its excellent compatibility with modular clinical workflows. Research objectives, data characteristics, and interpretability requirements jointly determine the choice of integration strategy. Enhancing the performance of these strategies depends heavily on advances in bioinformatics workflows and AI algorithms.

### 3.3 Application of bioinformatics and AI in multiomics integration

Bioinformatics provides the foundational framework for standardizing, functionally annotating, and mechanistically interpreting multiomics data, whereas AI technologies, with their powerful high-dimensional modeling and pattern-recognition capabilities, can overcome many limitations of traditional analytical methods. Together, they form the core technical infrastructure for multiomics integration. To address common challenges in multiomics data, such as platform-specific technical variation, high-dimensional sparsity, and data isolation, bioinformatics focuses on data quality control, standardization, and biological functional anchoring to ensure reliable and biologically meaningful inputs. AI, in turn, focuses on deep feature extraction, integrative modeling, and translational prediction, systematically addressing data heterogeneity and nonlinear relationships to improve feature selection accuracy and interpretability (Xu S. et al., 2024; Xu Z. et al., 2024). Multiomics integration typically begins with a bioinformatics-driven data normalization pipeline. During quality control, FastQC and Trimmomatic (Houmenou et al., 2025) can be used to assess read quality and remove adapter contamination and low-quality reads, while SAMtools and GATK perform sequence alignment and variant calling to ensure that raw data meet downstream analytical requirements. Subsequently, batch effects in raw count data (such as gene expression and methylation site counts) can be corrected with ComBat-seq, while domain-adversarial training can be applied to normalized continuous data (such as protein abundance and metabolite concentrations) to reduce platform differences (Xu F. Z. et al., 2023). In parallel, clusterProfiler can be used for GO/KEGG pathway enrichment analysis to map features onto biological pathways, removing redundant information unrelated to the biological processes of interest (Xu S. et al., 2024). During the initial feature selection, differential analysis can be performed using tools such as DESeq2 (Shafqat et al., 2025) and limma (Costa-Silva et al., 2017). Candidate features are then further reduced using machine learning methods such as LASSO regression and random forests, with nested cross-validation to minimize overfitting.

A major breakthrough in multiomics integration lies in the deep synergy between bioinformatics and AI. In the early stages, public resources, such as GWAS, mQTL, and eQTL datasets, can be integrated to provide prior biological knowledge for AI models (Dong et al., 2024). To address limited sample sizes, bioinformatics-based synthetic data generation techniques (e.g., variational diffusion models) can be used to simulate realistic multiomics data distributions and expand training sets, thereby alleviating data scarcity (Selvarajoo and Maurer-Stroh, 2024). On this basis, AI technologies achieve deep integration *via* several innovative pathways: (i) Using transformer architectures for multimodal feature fusion, leveraging self-attention to capture long-range dependencies between omics (e.g., regulatory relationships between methylation sites and miRNA expression), thus avoiding the dimensionality curse of simple concatenation (Choi and Lee, 2023). (ii) Introducing generative missing-value imputation methods such as MIWAE (Missing-Value Imputation with Wasserstein Autoencoders), which specifically address high dimensionality, heterogeneity, and missing data in multiomics while preserving key features and topological relationships, thereby improving data completeness (Cao et al., 2023). (iii) Adopting biology-driven modeling paradigms that map omics features onto protein–protein interaction networks (e.g., STRING) or metabolic pathway graphs, and applying graph neural networks (GNNs) to uncover nonlinear relationships among gene mutations, protein expression, and metabolic perturbations, thereby elucidating complex disease mechanisms at a systems level (Gogoshin and Rodin, 2023).

The clinical translation of these models depends critically on improved interpretability. SHAP (SHapley Additive exPlanations) analysis can quantify the contribution of each omics feature (Ponce-Bobadilla et al., 2024) and generate actionable decision rules, such as “trigger a high-risk alert when feature A exceeds threshold X and feature B exceeds threshold Y.” Coupled with the GO/KEGG pathway annotation, these rules provide mechanistic explanations that can be leveraged to enhance the sensitivity of early CRC detection. At the same time, federated learning frameworks support cross-institutional collaboration by sharing only model parameters, effectively mitigating the “data silo” problem. Synthetic data techniques employing differential privacy help protect patient confidentiality and comply with regulations such as HIPAA and GDPR (Calvino et al., 2024).

## 4 Clinical advances in multiomics liquid biopsy for colorectal cancer

### 4.1 Population screening and early diagnosis

Early CRC detection is crucial for reducing mortality; the 5-year survival rate for stage I patients exceeds 90%, whereas survival drops below 20% in advanced or metastatic disease even after systemic treatment (Siegel et al., 2023). However, current clinical early-screening strategies still face numerous bottlenecks. Conventional colonoscopy, although the diagnostic gold standard, requires complex bowel preparation and is invasive, resulting in low compliance among eligible populations. Traditional methods also struggle to capture molecular abnormalities at precancerous stages, leading to missed screening opportunities.

In recent years, multiomics liquid biopsy has emerged as a pivotal breakthrough for population-based CRC screening, owing to its non-invasive nature and high sensitivity. In 2014, the FDA approved Cologuard, a non-invasive test for CRC and precancerous lesions that combines fecal DNA analysis with hemoglobin detection, enabling the detection of KRAS mutations and BMP3 methylation (Zamanian et al., 2025). The Dxcover® liquid biopsy platform was validated in 957 patients from a U.S. multicenter cohort, achieving an AUC of 0.95 for CRC diagnosis with 90% sensitivity and 89% specificity; detection rates were 75% for stage I CRC and >93% for stages II–IV (Cameron et al., 2025). Mutation Capsule Plus technology innovatively integrates multiomics features, including DNA methylation, copy number variation, gene mutations, and 5′-end motifs, achieving a validation AUC of 0.981 (sensitivity 80% for stage I, 89.2% for stage II), outperforming single-omics genomic or epigenomic assays (Gao et al., 2024). cfDNA fragmentomics also demonstrates considerable potential; a stacking model built from low-depth whole-genome sequencing can predict early CRC with a sensitivity of approximately 90% (Chen et al., 2025). Technologies continue to advance; cfMeDIP-dPCR combines methylation detection with digital PCR, requiring only 0.5 mL of plasma to achieve CRC prediction with high sensitivity for KCNQ5, addressing the limitation of large sample volumes in traditional assays (Truong et al., 2025).

Cross-omics integration of transcriptomics with proteomics and the microbiome further expands screening capabilities. Tock et al. innovatively proposed hologenomic analysis using rectal mucus samples, which requires no bowel preparation and integrates host genomic, epigenomic, and microbial features to precisely differentiate adenomas from CRC, achieving superior stratification compared to single-omics host or microbiome tests (Tock et al., 2025). In saliva samples, combined detection of miR-29a (transcriptomics) with lactate and pyruvate (metabolomics) achieves an AUC of 0.89 and a compliance rate >90%, offering a novel scenario for non-invasive multiomics screening (Krokosz et al., 2025). Exosome-based subtype-specific detection is equally impressive: EVs secreted by CMS2-type CRCs are enriched for TSPAN8, whereas CMS4-type CRCs secrete TSPAN4-enriched EVs. The multidimensional combinations of mRNA, miRNA, and proteomic features carried by these subtype-specific EVs provide reliable evidence for precise CRC subtyping and screening (Asif et al., 2025).

### 4.2 Detection of minimal residual disease

MRD detection is a cornerstone of precision postoperative management of CRC, with clinical importance far surpassing traditional TNM staging and directly guiding treatment decisions and patient outcomes. Substantial clinical evidence confirms that postoperative MRD positivity is a strong predictor of recurrence; a meta-analysis of 3,568 patients showed a hazard ratio (HR) of 7.27 (95% CI 5.49–9.62) for recurrence-free survival in ctDNA-positive *versus* ctDNA-negative patients across all stages (Chidharla et al., 2023). This prognostic value has now been translated into therapeutic decision-making in landmark prospective, multicenter trials. In the DYNAMIC-III study, a phase 2/3 randomized trial enrolling 1,002 patients with stage III colon cancer across sites in Australia, Aotearoa New Zealand and Canada, post-operative ctDNA status at 5–6 weeks was used to guide adjuvant chemotherapy intensity. ctDNA-negative patients had a 3-year recurrence-free survival (RFS) of approximately 87%, compared with about 49% in ctDNA-positive patients, confirming ctDNA as a powerful predictor of molecular residual disease. Importantly, ctDNA-guided management reduced oxaliplatin use from nearly 90% to about 35% while maintaining similar 3-year RFS and lowering severe treatment-related adverse events, demonstrating that ctDNA can safely de-escalate adjuvant chemotherapy in patients without detectable MRD while focusing intensive treatment on those at highest risk of recurrence (Tie et al., 2025). The CIRCULATE-Japan GALAXY study, with 23 months of follow-up, confirmed that postoperative ctDNA-positive (MRD+) patients have approximately 12-fold and 10-fold increased risks of recurrence and death (DFS HR = 11.99, P < 0.0001); meanwhile, MRD-negative patients exhibit significantly better outcomes, especially those with sustained ctDNA clearance after adjuvant chemotherapy, achieving a 24-month disease-free survival rate of 89% (Nakamura et al., 2024). In current clinical practice, tumor-informed ctDNA assays such as Signatera™ (Natera) and RaDaR™ (NeoGenomics/Inivata) are offered as laboratory-developed tests that have received FDA Breakthrough Device Designation but are not yet FDA-cleared or approved. Signatera has obtained broad Medicare coverage in multiple cancer types and is currently under PMA review as a companion diagnostic, whereas RaDaR has been commercially launched as a highly sensitive MRD assay with payer coverage in selected markets (Abdo et al., 2026).

Unlike conventional imaging, which detects only visible lesions, MRD markers, such as ctDNA, can provide early warning of recurrence. In a prospective multicenter study of Vietnamese patients with CRC, interim analysis after 16 months of follow-up showed that ctDNA detected recurrence in two patients 4–10.5 months earlier than clinical diagnosis; CEA remained unchanged in both cases, highlighting the value of ctDNA as a key tool for overcoming CRC recurrence and improving survival (Nguyen et al., 2022).

Multiomics liquid biopsy, leveraging its non-invasive nature, dynamic monitoring capability, and multidimensional signal coverage, offers an ideal technical solution for MRD detection. Compared with the single-point sampling limitation of tissue biopsies, liquid biopsy can capture tumor-derived genomic, epigenomic, transcriptomic, and metabolomic signals circulating in the blood, effectively avoiding detection biases caused by tumor heterogeneity. In terms of technical performance, tumor-informed ctDNA assays (HR = 8.66) significantly outperform tumor-agnostic methods (HR = 3.76) for predicting postoperative recurrence. Although the latter shows statistical significance, it has weaker prognostic stratification power, indicating that personalized detection strategies confer greater clinical benefits (Chidharla et al., 2023). Markers from different molecular dimensions can complement each other; for instance, the epigenetic marker methylated SEPT9 (mSEPT9) has a positive detection rate of 82.6% in stage III CRC, which is significantly higher than that of CEA (60.3%, P < 0.001), and can effectively stratify recurrence risk before neoadjuvant therapy, whereas CEA lacks predictive value at this stage (Xu et al., 2025). Combining such high-sensitivity epigenetic markers with ctDNA could enhance MRD detection sensitivity in low tumor-burden settings, reducing false-negative rates. Moreover, liquid biopsy supports continuous postoperative monitoring without repeated invasive sampling, ideally meeting the clinical need for long-term MRD surveillance. Besides early detection and MRD, liquid biopsy also enables real-time therapy selection by detecting evolving resistance-conferring alterations and genetic reversion events. By dynamically tracking these molecular changes through serial ctDNA profiling, clinicians can adjust targeted or systemic therapies in a timely manner to overcome emerging resistance and avoid ineffective treatment (Bartolomucci et al., 2025).

**FIGURE 3:**
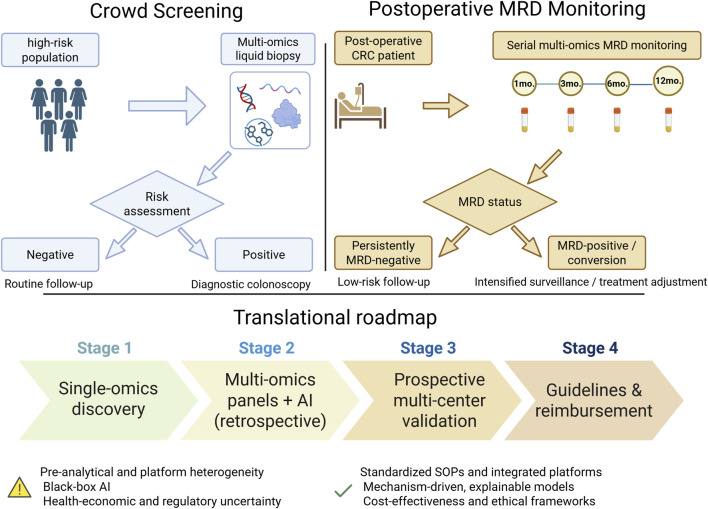
Clinical application pathways and translational roadmap of multiomics liquid biopsy in colorectal cancer. Multiomics liquid biopsy in colorectal cancer: schematic clinical pathways and translational roadmap. Upper panels show population based screening (left) and post operative minimal residual disease (MRD) monitoring (right). In screening, multiomics liquid biopsy is applied to high-risk populations to stratify individuals into low-risk routine follow-up *versus* positive cases referred for diagnostic colonoscopy. In the post-operative setting, serial multiomics profiling of blood samples (tumor-informed ctDNA combined with selected methylated DNA and RNA markers) is used to determine MRD status and guide follow-up intensity or treatment adjustment. The lower panel summarizes the stepwise translation from singleomics discovery to multiomics panels with AI, prospective multi-center validation, and eventual incorporation into clinical guidelines and reimbursement, highlighting key challenges and potential solutions.

Current MRD research in CRC shows a pronounced single-omics trend, with ctDNA-centric genomic assays dominating the field, whereas studies involving other omics are relatively scarce and fragmented. ctDNA has become the “gold standard” for MRD detection, supported by substantial clinical evidence; case reports show that ctDNA dynamic monitoring can promptly reveal treatment failure and guide transitions to immunotherapy for molecular remission (Burley et al., 2024). Moreover, patients with persistent ctDNA clearance after adjuvant chemotherapy achieve a 24-month DFS rate of 89.0%, compared with 3.3% for those with transient clearance (Nakamura et al., 2024). However, MRD research using other omics techniques remains underdeveloped. At the epigenomic level, mSEPT9 is currently the most robustly validated MRD marker, with postoperative positivity significantly predicting recurrence risk (HR = 6.21, P < 0.001) (Yuan et al., 2022). At the transcriptomic level, circulating miR-155, miR-221, miR-34a, and miR-143 show dynamic changes after CRC surgery, and their combination can distinguish patients from healthy individuals with high precision (AUC = 0.999), serving as a promising non-invasive biomarker for early tumor clearance (Ham-Karim, 2025). Metabolomic studies have also advanced; Zhou et al. (Zhou Z. et al., 2025) built a machine-learning model incorporating multiple metabolites, demonstrating the ability to predict CRC recurrence up to a median of 471 days before clinical detection, with 62% sensitivity and 80% specificity, indicating its potential as a novel MRD detection tool. The limitation of current studies lies in their single-omics and fragmented nature, which hampers their ability to meet the complex clinical demands of MRD detection. Most studies focus on single markers within a single omics layer, lacking multidimensional integrated models, which restricts further improvement in detection sensitivity, specificity, and stability, and cannot meet the stringent requirements of MRD detection under low tumor burden. Therefore, strengthening multiomics integration is essential for overcoming current MRD detection bottlenecks. Future efforts should use ctDNA as the core framework and systematically integrate epigenomic, transcriptomic, and metabolomic signals to construct standardized combined detection models. Multicenter, large-scale validation studies are needed to confirm clinical efficacy, standardize workflows and interpretation criteria, and fully leverage the technological advantages of liquid biopsy, thereby overcoming the inherent limitations of single-omics approaches and providing more precise, reliable clinical tools for CRC MRD detection, ultimately reducing postoperative recurrence rates and improving patient outcomes (Figure 3).

## 5 Clinical translation bottlenecks of multiomics liquid biopsy

Despite providing unprecedented molecular insights into early CRC detection and dynamic monitoring, multiomics liquid biopsy faces multiple systemic barriers before transitioning from research to routine clinical practice (De Paolis et al., 2025). These challenges arise not from isolated technical flaws but from the complexity of multimodal data integration, clinical validation pathways, and translational ecosystems, demanding interdisciplinary collaboration.

The dimensions captured by different omics layers differ markedly; genomic mutations are highly specific but present at very low frequencies (Newman et al., 2014). Epigenetic markers (e.g., mSEPT9) are stable but lack absolute tissue specificity (Ransohoff, 2021). Transcriptomic and metabolomic signatures are dynamic but susceptible to inflammation, diet, and circadian rhythms (Wang et al., 2024). More critically, the kinetic characteristics of these biomarkers vary; ctDNA has a half-life of ∼1–2 h (Diehl et al., 2008; Khier and Lohan, 2018), whereas some methylation fragments or metabolites persist for days or longer. This pronounced spatiotemporal heterogeneity makes it difficult to capture all key signals simultaneously in a single sample, and longitudinal monitoring may lead to false negatives or overinterpretation owing to mismatched timing windows. Additionally, for early-screening applications, multiomics strategies must address false-positive results, which can lead to overtreatment and psychological distress; cost-effectiveness at the population level remains poorly quantified, introducing significant uncertainty from a health-economic perspective.

Simultaneous multiomics profiling from a single biospecimen faces three major practical and technical constraints that go beyond analytical data integration. First, sample volume requirements increase substantially: to obtain sufficient cfDNA/cfRNA, extracellular vesicles, proteins and metabolites for parallel assays, approximately 5–10 mL of plasma (or an equivalent urine volume) are often needed (Pandey and Yadav, 2025). This is challenging in patients with limited venous access, in screening settings requiring repeated sampling, and in vulnerable groups such as the elderly or those with anemia. Second, different analyte classes have incompatible pre-analytical handling and cold-chain needs: cfDNA/ctDNA requires prompt plasma separation in EDTA tubes and rapid freezing to avoid leukocyte lysis and genomic DNA contamination; cfRNA relies on RNA-stabilizing reagents and strict low-temperature control to maintain integrity; protein biomarkers typically need protease inhibitors and minimized freeze–thaw cycles; whereas metabolites are best preserved by immediate freezing and long-term storage at low temperatures (e.g., −80 °C). These divergent requirements complicate unified sample processing and increase the risk of pre-analytical bias across centers. These divergent requirements substantially complicate unified sample processing and increase the risk of pre-analytical bias across centers. (Grölz et al., 2018; Sarathkumara et al., 2022; Simon et al., 2025; Van Der Schueren et al., 2025). Third, truly integrated multiomics workflows demand substantial infrastructure, including next-generation sequencing platforms, high-resolution mass spectrometers, automated liquid-handling systems and dedicated bioinformatics support, which are usually confined to centralized reference laboratories rather than point-of-care environments. As a result, cold-chain transport, platform variability and site-specific pre-analytical workflows introduce batch effects and domain shift in data distributions, posing a major challenge to the robustness and generalizability of AI-driven liquid biopsy models in real-world deployment (Hsu et al., 2025).

To maximize predictive potential, current studies widely employ machine learning or deep learning strategies to integrate high-dimensional features. Although these models demonstrate impressive statistical performance, most are “black-box” algorithms whose outputs cannot be directly linked to specific biological mechanisms or actionable therapeutic targets. In clinical decision-making, physicians need not only to answer “Is this patient at high risk for recurrence?” but also “Which pathways or molecules drive that risk assessment?” and “What intervention should follow?” Without mapping model outputs to actionable pathways or treatment targets, clinical trust and adoption are substantially compromised. Therefore, future work must develop integrative algorithms that combine performance with interpretability (Klauschen et al., 2024), such as pathway enrichment or multiomics co-regulatory network modeling strategies.

In summary, the clinical translation of multiomics liquid biopsy in CRC is constrained by three interrelated bottlenecks. First, pronounced spatiotemporal heterogeneity across genomic, epigenomic, transcriptomic and metabolomic layers makes it difficult to capture all key signals in a single sample and increases the risk of false negatives or overinterpretation in longitudinal monitoring. Second, the lack of standardized pre-analytical and analytical workflows and the presence of cross-platform “data silos” (e.g., NGS, qPCR and MS) lead to poor reproducibility and limit cross-center validation and regulatory acceptance. Third, most current AI-based multiomics models still function as “black-box” systems that provide limited mechanistic interpretability and are not yet supported by systematic clinical and health-economic evaluation, undermining clinical trust and large-scale implementation. (Klauschen et al., 2024).

## 6 Future outlook and development directions

The true value of multiomics in CRC liquid biopsy does not lie in adding more data, but in its ability to address clinical scenarios that single-omics approaches cannot tackle. As summarized in Section 5, its clinical translation is currently hampered by data heterogeneity, lack of standardized workflows and limited interpretability of AI-based models. From concept validation to clinical translation, future research should focus on technical standardization, deep integration of multiomics models, prospective clinical trial validation, and health-economic assessment. Commercial and near-market assays (Table 2) have demonstrated the scalability of multi-analyte blood-based testing, but also underscore persistent limitations, including modest sensitivity in early-stage or low-shedding cancers, reliance on centralized high-throughput platforms, and uncertain cost-effectiveness in population-based programs. The multiomics frameworks proposed in this review—standardized integrated workflows, mechanism-driven interpretable models, and prospective multi-center validation—are designed to address these real-world barriers and to improve both the performance and generalizability of next-generation CRC-focused assays.

**TABLE 2:** Representative commercial or near-market multiomics liquid biopsy assays.

Assay/company	Analyte compositionandplatform	Intend clinical use	Regulatory status	Key limitation
Cancerguard™ (Exact Sciences; evolved from CancerSEEK)	Plasma cfDNA methylation + a panel of tumor-associated proteins; multiplex immunoassays combined with multi-biomarker integration	Multi-cancer early detection in asymptomatic adults (50+ cancer types)	Laboratory-developed test under CLIA; not yet widely reimbursed for population screening	Moderate overall sensitivity and reduced performance in stage I disease; limited data in low-shedding tumors and organ-specific MRD settings
Galleri® (GRAIL)	Plasma cfDNA methylation patterns at >100,000 informative loci; targeted bisulfite sequencing	Multi-cancer early detection in average-risk or elevated-risk adults	CLIA-regulated LDT; large prospective trials ongoing; not FDA-approved as a primary screening test	Overall sensitivity ∼50% with relatively low sensitivity for stage I cancers; unclear impact on cancer-specific mortality; little evidence in CRC-specific MRD monitoring
SimpleScreen™/Freenome	Multiomics platform combining genomic and epigenomic features in cfDNA with circulating protein signals, integrated by AI/ML	Blood-based colorectal cancer screening and detection of advanced precancerous lesions	Premarket approval submission to FDA ongoing; not yet approved for routine screening	Requires relatively large plasma volumes and complex centralized workflows; early-stage lesion sensitivity remains modest, and performance in post-operative MRD is unknown
Shield™ CRC test (Guardant Health)	Plasma cfDNA alterations integrating somatic mutations, methylation and fragmentation features *via* hybrid-capture NGS	Primary blood-based screening test for colorectal cancer in average-risk adults	FDA-approved blood test for CRC screening; covered by several payers	Validated mainly for prevalent CRC; shows limited sensitivity for advanced adenomas and is not yet evaluated as a multiomics MRD assay

Table 2 summarizes representative assays, including their analyte composition, intended clinical use and regulatory status, together with key performance characteristics and known limitations. Although most of these products are currently developed for pan-cancer screening rather than CRC-specific MRD monitoring, their real-world experience provides important insights into the practical challenges and potential impact of multiomics liquid biopsy at scale.

In response to these challenges, future development should prioritize establishing comprehensive standards that span the entire workflow of liquid biopsy. These should include standardized protocols for processing cfDNA, cfRNA, extracellular vesicles, and metabolites in plasma, urine, feces, and other biofluids to minimize pre-analytical variability. There is an urgent need to develop integrated platforms capable of parallel capture of genomic, epigenomic, transcriptomic, and proteomic/metabolomic signals from minimal sample volumes. GAO et al. demonstrated an integrated approach using MCP technology, combining DNA methylation, copy number variation, gene mutations, and 5′-end motifs to achieve 80% sensitivity for stage I CRC, providing an important example of multiomics integration (Gao et al., 2024).

Future multiomics integration should shift toward mechanism-driven modeling anchored in core CRC pathways. Building on the limitations of current “black-box” models outlined above (Section 5), future work is increasingly embedding prior knowledge, such as the Wnt/β-catenin and EGFR/MAPK pathways, or large biological databases, into AI architectures. For example, DeePathNet uses a transformer framework combined with cancer pathway databases for joint modeling to enhance the prediction accuracy for cancer subtypes and drug responses (Cai et al., 2024). In addition, developing subtype-specific multiomics panels based on CMS molecular classification will enable truly individualized monitoring and intervention (Asif et al., 2025).

The core advantage of multiomics liquid biopsy is its ability to significantly enhance the detection of CRC and precancerous lesions, as well as the sensitivity of MRD monitoring through multidimensional signal synergy. However, to transition from technical validation to population screening and routine clinical use, future studies must systematically evaluate clinical utility, health economics, ethics, and societal impact (Osti et al., 2025). From a health-economic perspective, cost-effectiveness ratios in real-world settings should be quantified. Although initial costs are higher, multiomics testing could lead to more efficient resource allocation in the medium to long term if it increases early detection rates, reduces unnecessary colonoscopies, and optimizes postoperative follow-up, thereby lowering overall healthcare expenditure and extending high-quality survival time. Simultaneously, prospective studies on data privacy, informed consent, result interpretation, and psychosocial impacts are required to establish appropriate governance frameworks. Only by balancing technological performance, economic sustainability, and social acceptability can multiomics liquid biopsy be transformed into a valuable public-health tool for CRC prevention and control.

## 7 Conclusion

Multiomics liquid biopsy, through the integration of multi-layered circulating biomarkers, significantly enhances the early detection of CRC and precancerous lesions and the sensitivity of MRD monitoring. However, its clinical translation remains limited by insufficient technical standardization, limited depth of data integration, and a lack of health-economic evidence. Future efforts must focus on integrated platform development, mechanism-driven explainable model design, and systematic evaluation of cost-effectiveness and social acceptability in real-world screening and monitoring to fully realize its public-health value.
